# The Experience of Beauty of Chinese Poetry and Its Neural Substrates

**DOI:** 10.3389/fpsyg.2018.01540

**Published:** 2018-08-21

**Authors:** Chunhai Gao, Cheng Guo

**Affiliations:** ^1^Laboratory of Personality Development and Social Adaptation, Department of Psychology, Southwest University, Chongqing, China; ^2^Research Center of Mental Health Education, Southwest University, Chongqing, China

**Keywords:** Chinese poetry, reading, appreciation, beauty, OFC, insula, neuroesthetics, fMRI

## Abstract

Chinese poetry has a long history and high esthetic value. People who engage esthetically with Chinese poetry would feel the sense of beauty naturally. However, there is little information regarding what happens in the brain when an individual appreciates Chinese poetry, and how the brain processes the subject’s appreciation of beauty. Herein, we used functional magnetic resonance imaging (fMRI) to investigate the neural substrates of experiencing beauty by appreciating Chinese poetry. The participants in our study were 28 college students and the stimuli consisted of 25 Chinese poetry and 25 prose selections. Based on an event-related paradigm, the findings of this study suggested that different areas scattered in both the left and right cerebral hemispheres are activated when an individual appreciates Chinese poetry. Compared to reading prose, appreciating Chinese poetry heightens the activation of the left inferior orbitofrontal cortex (OFC), the bilateral insula, the left fusiform, the left supplementary motor area (SMA), and the left precentral gyrus. In these areas, the left inferior OFC and the bilateral insula are considered closely related to experiencing beauty of Chinese poetry, which have been demonstrated that it is an important neural basis of esthetic beauty when using other types of materials. The findings of this study shed new light on the complex but ordinary processes of experiencing beauty when appreciating Chinese poetry and show that some key processes underlying the feeling of esthetic beauty are shared across different esthetic domains.

## Introduction

Individuals who appreciate poems can feel a sense of beauty while doing so. In fact, this is one reason that they read poems. This has already been established in studies carried out at different times and in different cultures, as well as in individuals of all ages. Chinese poetry, which have high esthetic value, comprise an important form of poetry. Written forms of Chinese poetry have been found to exist as early as the first millennium BC. These poems were written based on the daily working lives of individuals, especially those involved in singing and dancing. Chinese poetry are representative of Chinese culture, and are concentrated expressions of Chinese art. Large numbers of Chinese individuals enjoy reading Chinese poems. In addition, these poems are popular among individuals who like poetry and have interest in Chinese culture. In general, like other forms of poetry, Chinese poetry is naturally associated with the expression and inspiration of affective and impressive meanings and emotions (Ludtke et al., 2014). The terms and speech used in Chinese poetry are esthetically and perceptually appreciated (Schrott and Jacobs, 2011). However, the manner in which the brain processes Chinese poetry is unclear. In addition, the specific neural substrates for the feeling of beauty stimulated by these brain activities are still unknown. We investigated the neural substrates of the feelings induced by appreciating Chinese poetry and determined the regions whose activities are correlated with feelings of beauty. Investigating the activation of brains stimulated by the esthetic appreciation of Chinese poetry is of great value. First, it is important for the humanities to understand the problem of esthetic appreciation of poems at a basic level. Second, our investigation provides evidence for the cross-cultural consistency of the esthetic appreciation of poems. Third, our investigation is helpful in understanding human esthetics, and especially the neural basis of esthetics. Our research findings also provide empirical evidence that may be used to study the neural mechanisms of poetic esthetics.

Advances in non-invasive neuroimaging techniques have allowed us to research healthy participants under controlled situations, and to associate the appreciation of the beauty of poetry with the activities of several brain structures. We can also use neuroimaging to study the processes of underlying the appreciation of beauty in art forms other than poetry. The field of neuroesthetics has thus been developed to answer questions regarding the neural underpinnings of esthetics (Pearce et al., 2016). New theoretical work and experimental studies have led to remarkable progress in neuroesthetics.

Neuroimaging studies have been used to investigate the neurocognitive underpinnings of the esthetic appreciation of different art forms, such as paintings (Vartanian and Goel, 2004; Cupchik et al., 2009; Kirk et al., 2009b), sculptures (Di Dio et al., 2007), architecture (Kirk et al., 2009a), everyday designed products (Yeh et al., 2015), human faces (Aharon et al., 2001; Ishai et al., 2007; Winston et al., 2007), abstract geometrical patterns (Jacobsen et al., 2006), mathematics (Zeki et al., 2014), music (Blood and Zatorre, 2001; Koelsch, 2010; Ishizu and Zeki, 2011; Brattico and Pearce, 2013; Zatorre and Salimpoor, 2013; Koelsch and Skouras, 2014), dance (Calvo-Merino et al., 2005, 2008; Cross and Ticini, 2012), and literature (Bohrn et al., 2013). Research in the different esthetic domains listed above has thus far been unbalanced. The majority of empirical studies have been carried out to investigate visual and auditory domains, while research in other fields, such as literature appreciation, is less common.

Although relevant empirical studies are relatively few, some representative research has been reported. Zeman et al. (2013) studied brain activation by poetry and prose. O’Sullivan et al. (2015) studied the neural basis of literary consciousness. Bohrn et al. (2013) performed pioneering research in this field when they attempted to determine whether reading is accompanied by an implicit esthetic evaluation. In the experiments described in the above report, the participants were requested to read a number of proverbs without explicitly evaluating them. The authors found that large parts of the left frontal lobe, left medial temporal gyrus, left superior temporal gyrus, bilateral occipital lobes, and bilateral precentral gyri were activated during the reading activities. They also identified correlations between specific brain regions and feelings of beauty. These regions were the right caudate nucleus, the anterior cingulate, and the cerebellum. On the basis of empirical research results, Jacobs (2015) proposed a model called the neurocognitive poetics model (NCPM) to elucidate the mechanisms of poetry reception using a neural perspective. The NCPM postulates that different features of poetry texts can activate different neural networks and cognitive-affection processes.

Stimuli other than appreciating literature have also been found to have neural correlates of the feeling of beauty. Kawabata and Zeki (2004) found that the orbitofrontal cortex (OFC) is differentially engaged during the viewing of beautiful paintings. Previous research using other stimuli in the visual esthetic domain (Kirk, 2008; Kirk et al., 2009a,b; Lacey et al., 2011; Ishizu and Zeki, 2013; Flexas et al., 2014; Zhang et al., 2016) and the music esthetic domain (Ishizu and Zeki, 2011) has led to similar conclusions. The insula is another key brain region involved in feelings of beauty. This reflects the “viscerality” of esthetic perception (Cupchik et al., 2009; Brown et al., 2011; Flexas et al., 2014). Jacobs et al. (2012) found that the frontomedial cortex and the amygdala appear to be selectively sensitive to beauty during beauty judgments. In addition, the lingual gyrus has been shown to be activated in some studies of beauty (Kawabata and Zeki, 2004; Vartanian and Goel, 2004; Mizokami et al., 2014; Vartanian and Skov, 2014). There are no consistent conclusions regarding the brain regions that are important for the feeling of beauty thus far.

In sum, the main purpose of our experiment was to investigate the neural substrates of the experience of beauty induced by appreciating Chinese poetry. Changes in regional cerebral blood flow can be measured during the appreciation of Chinese poetry to test our hypothesis. Our study was based on the following hypotheses:

H1. *The basis of the esthetic appreciation of Chinese poetry is similar to that of German proverbs and English poems.*

Brain activity during the appreciation of literature has been investigated using other types of literary material, such as German proverbs (Bohrn et al., 2013) and English poems (Zeman et al., 2013; O’Sullivan et al., 2015). However, the brain activity induced by the appreciation of Chinese poetry is still unknown. We expect that there is some consistency in the neural basis of the effects of appreciating literature in any type or form.

H2. *Comparison of brain activity patterns observed during the appreciation of Chinese poetry to those observed during the reading of prose will reveal significant activation patterns in specific brain regions (e.g., OFC and insula) that are related to the appreciation of beauty. This is in accordance with previous research on the appreciation of beauty in the visual and auditory arts.*

Previous studies on the esthetic appreciation of visual and auditory arts have shown that the feeling of beauty may recruit cortical systems such as the OFC (e.g., Kawabata and Zeki, 2004; Kirk, 2008; Kirk et al., 2009a,b; Lacey et al., 2011; Ishizu and Zeki, 2013; Flexas et al., 2014; Zhang et al., 2016) and insula (e.g., Cupchik et al., 2009; Brown et al., 2011; Flexas et al., 2014). Therefore, we hypothesize that comparing brain activity patterns observed during the appreciating Chinese poetry to those observed during the reading of prose can reveal significant activation in brain regions related to the appreciation of beauty induced by other art forms, especially in the OFC and the insula. Differences in brain activation induced by the reading of poetry vs. prose have previously been studies (Zeman et al., 2013; O’Sullivan et al., 2015). However, there are no consistent conclusions regarding this issue. Our study should validate the results obtained by other researchers and identify brain regions associated with the esthetic of poetry. The neural correlates of the appreciation of beauty induced by Chinese poetry should recruit the cortical system in a similar manner to those of the much better investigated esthetic appreciation of other art forms.

## Materials and Methods

### Participants

Twenty-eight right-handed participants (mean age: 19 years; range: 18–21 year; 14 women and 14 men) who were healthy and had normal vision or corrected-to-normal vision participated in this study. The participants were good at reading^1^. Bad readers (e.g., those with dyslexia) were excluded. None of the participants had received special poetry education and all were naive to the hypothesis of this experiment. All participants voluntarily agreed to participate in this study and signed a written informed consent prior to their inclusion in the study. The study was supported and sponsored by the Human Ethics Committee of the Southwest University for the Brain Mapping Research. The participants received financial rewards at the end of the study.

### Stimuli

**FIGURE 1:**
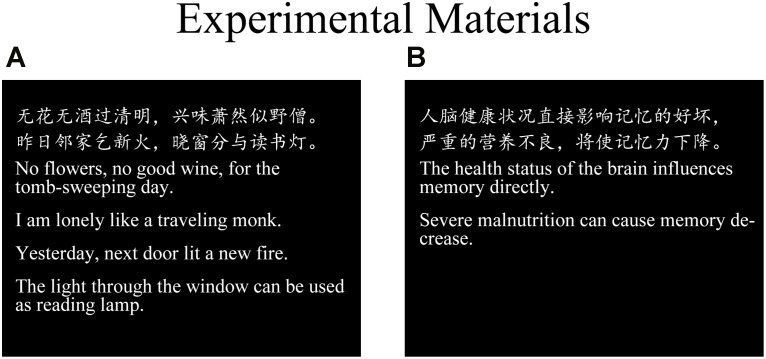
Examples of experimental stimuli. **(A)** An example of a Chinese poetry. **(B)** A prose selection.

Two types of stimuli were used: poetry and prose. Each group of stimuli comprised 25 items (samples of the materials are shown in **Figure 1**). The stimuli shown to the participants were identical. All of the selected poems were popular *Qi Yan Jue Ju* (*Qijue* in short; i.e., seven-character quatrain), which is a type of famous Chinese poetry. In order to better study and research the neural substrates of esthetic beauty induced by appreciating Chinese poetry, high-quality poems were chosen first. High-quality Chinese poetry has the following features:

(1) The language of the poetry is succinct and the wording of the poetry is fastidious.
(2) The poetry displays abundant and multiple artistic conception tastes.
(3) The rhythms of the poetry are strictly in accordance with the standards of Chinese poetry.
(4) There are only few words that are rarely used in poetry.
(5) The poetry is liked by individuals from different times.

There are many different types of high-quality Chinese poetry. In this study, we chose *QiJue* as our experimental stimuli for three reasons. First, *Qijue* has high artistic value, which individuals appreciate. Second, *Qijue* has a very formal style. *Qijue* has strict requirements for sentence number in each poem and for character number in each sentence. There are generally four sentences in each poem and seven characters in each sentence. Third, *QiJue* is marked by strict tonal patterns and rhyme schemes constituting a well-structured prosodic hierarchy (Li and Yang, 2010). Because of this feature, we were able to ensure that all poems had the same number of words, sentence length, tonal patterns, rhythm, and rhyme. The poems used in the experiment were chosen from *Chinese Literature Education* and were equally familiar to all participants. The prose selections, which served as the control condition stimuli, lacked poetry characteristics and stylistic features, but had valid literal interpretations. The topics of the carefully chosen prose selections were familiar to the participants (simple statements regarding world-knowledge). Lexical parameters, such as the number of words and sentence length, were matched across the prose selections and were identical to those of the poems. The font type, font color, font size, and line spacing were consistent in all of the texts.

### Procedure

The trials were presented using an event-related design while the participants were in the scanner. The Chinese poetry and the prose selections were randomly sorted and appeared in succession. In order to directly compare our results to those of researchers who have explored brain activity correlated with beauty, using other types of stimuli (Vartanian and Goel, 2004; Jacobsen et al., 2006; Winston et al., 2007; Kirk et al., 2009b), we decided to adopt experimental procedures similar to those used in the above studies. While we based our procedures on the above research, we made some improvements to the procedures in our study. Chinese poetry and prose were read during the scan. Similar functional magnetic resonance imaging (fMRI) research regarding the appreciation of Chinese poetry has not been conducted before. Therefore, a behavioral pre-experiment was designed to test the durations of the stimuli. We found that the average time required for the participants to appreciate a poem sufficiently was 11.33 s. We therefore used 12 s as the reading time in the fMRI experiment. After reading and appreciating the stimuli, the participants were required to provide an explicit esthetic judgment. Our experiment was thus different from a previous study that used an implicit esthetic judgment (Bohrn et al., 2013). We chose to include an explicit esthetic judgment task during the fMRI because it was likely to enhance the effects of the stimuli by requiring the participants to pay attention to the esthetic qualities of the stimuli. This task also increased the possibility that the participants would engage in the more advanced cognitive stages of the interpretation and evaluation of art-specific attributes despite the wide range of other processes that may subconsciously impact the esthetic experience (Leder et al., 2004). Furthermore, the time required to appreciate Chinese poetry was obviously longer than that required to read a proverb. This also contributed to our decision to choose an explicit esthetic judgment task.

Before entering the scanner, the participants received complete instructions regarding the research, a consent form, and a personal information form. A brief practice session was also carried out. Once inside the scanner, the participant’s head was fixed using foam pads, which minimized the motions of the participant’s head. The stimuli were displayed on a back-projection screen positioned at the bottom of the scanner. The stimuli were viewed via a mirror placed on top of the head coil. The responses of the participants were recorded using two fiber-optic button boxes tied to the participant’s right and left hands. E-Prime (Psychological Software Tools, Inc., Sharpsburg, PA, United States) was used to present and time the stimuli, and to collect data.

**FIGURE 2:**
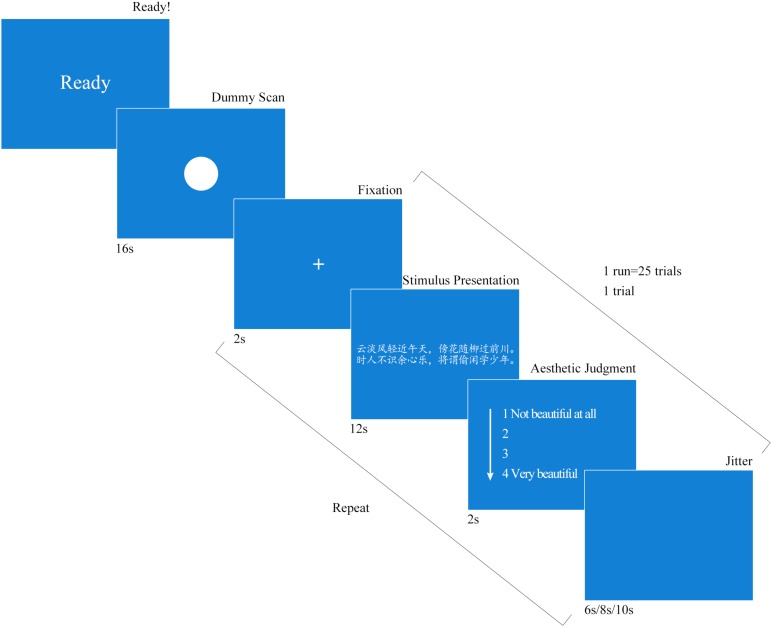
Experimental design. The experiment was performed in two runs. Twenty-five stimuli were presented in each run.

Our experiment consisted of two runs, each of which consisted of 25 trials. Each run started with a display of the word “ready” on the screen, which was followed a dummy scan for 16 s. The 25 trials were then presented consecutively. Each trial was initiated by a 2-s display of a fixation cross at the center of the viewing screen and was followed by a stimulus. The stimulus was presented for 12 s. During the presentation of the stimulus, the participants were asked to focus on appreciating the stimulus in silence. After appreciating the stimulus, the participants were requested to complete an esthetic judgment task, which consisted of rating the beauty of the stimulus on a scale ranging from 1 (no sense of beauty) to 4 (great sense of beauty). The participants were asked to provide their ratings within 2 s. Two fiber-optic button boxes were held in the participants’ right and left hands. Each button box had two buttons. The buttons represented 1, 2, 3, and 4, respectively. Half of the participants used their right hands to press 1 and 2, and the other half used their left hands to press 1 and 2 when entering their answers. A blank screen was displayed after the rating task. The inter-trial intervals were randomly chosen to last 6, 8, or 10 s. Each run lasted for approximately 616 s. Every subject took part in both runs. There was a 3-min break between the two runs. **Figure 2** illustrates the task and the procedures.

### fMRI Acquisition

Whole-brain functional data were acquired using a 3T Siemens scanner (Siemens Magnetom Trio TIM, Erlangen, Germany). During the visual presentations, blood oxygen level-dependent imaging was performed using a single-shot echo-planar imaging sequence with the following parameters: interslice skip = 0.99 mm; number of slices = 32; repetition time = 2,000 ms; echo time = 30 ms; flip angle = 90°; field of view = 220 mm × 220 mm; matrix size = 64 × 64; voxel size = 3.4 mm × 3.4 mm × 3 mm. Structural data were acquired using T1-weighted images recorded from 176 1-mm-thick slices with an in-plane resolution of 1 × 1 mm (repetition time = 1,900 ms; echo time = 2.52 ms; flip angle = 9°; field of view = 250 mm × 250 mm).

### Data Analysis

Functional images were preprocessed using SPM8^2^, which was implemented in MATLAB 2014 (Mathworks Inc., Sherborn, MA, United States). The first five images were discarded to achieve steady magnet states. For T2^∗^-weighted images, the slice order was corrected through slice timing, and head movements > 2 mm or 2° were removed. The T1-weighted images were co-registered to the mean echo-planar images and segmented into white matter, gray matter, and cerebrospinal fluid. The echo-planar images were then normalized to the Montreal Neurological Institute space using structural information obtained during the co-registration and segmentation. The voxel size was 3 mm× 3mm × 3 mm. Spatial smoothing was performed using a Gaussian kernel of 8 mm × 8 mm × 8 mm at full width at half maximum.

As the first-level analysis, a general linear model was applied to the fMRI time-series wherein stimulus onset was modeled as a single impulse response function. The data were then convolved with the canonical hemodynamic response function. We modeled two conditions of interest: poetry and prose. Head movement parameters were calculated based on the realignment procedure, and were included in the model as covariates of no interest. A high-pass filter with a cutoff of 128 s was used to remove low-frequency signal drifts. As the second-level analysis, activation patterns induced by appreciating Chinese poetry were obtained using a one-sample *t*-test. We used the contrast between appreciating poetry and reading prose to identify brain regions related to feelings of beauty induced by the appreciation of Chinese poetry (poetry > prose). False discovery rate-corrected *p* values < 0.05 were considered significant. A cluster size threshold of 40 was used.

## Results

### Behavioral Results

**FIGURE 3:**
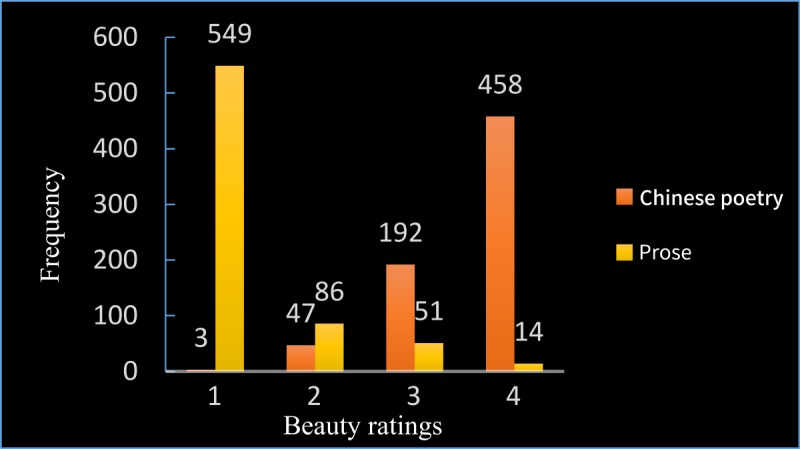
Frequencies of beauty scores. The participants were requested to rate the stimuli on a beauty scale after reading the Chinese poetry or prose selections. The scale ranged from 1 to 4. A score of 1 indicated no sense of beauty, 2 indicated a weak sense of beauty, 3 indicated a fair sense of beauty, and 4 indicated a great sense of beauty.

The mean esthetic judgment rating scores for the Chinese poetry and the prose selections were 3.58 ± 0.64 and 1.33 ± 0.70, respectively. The frequencies of the beauty ratings of the poetry and the prose selections by the participants are shown in **Figure 3**. The Chinese poetry had high beauty ratings, while the prose selections had low beauty ratings. Comparisons between the ratings of the Chinese poetry and those of the prose selections indicate that participants reading Chinese poetry provided significantly higher ratings than those reading prose (*t* = 61.49, *p* < 0.0001, Cohen’s *d* = 3.35). Participants who read Chinese poetry felt more beauty than those reading prose.

### fMRI Results

#### Areas of Brain Activation During the Appreciation of Chinese Poetry

**FIGURE 4:**
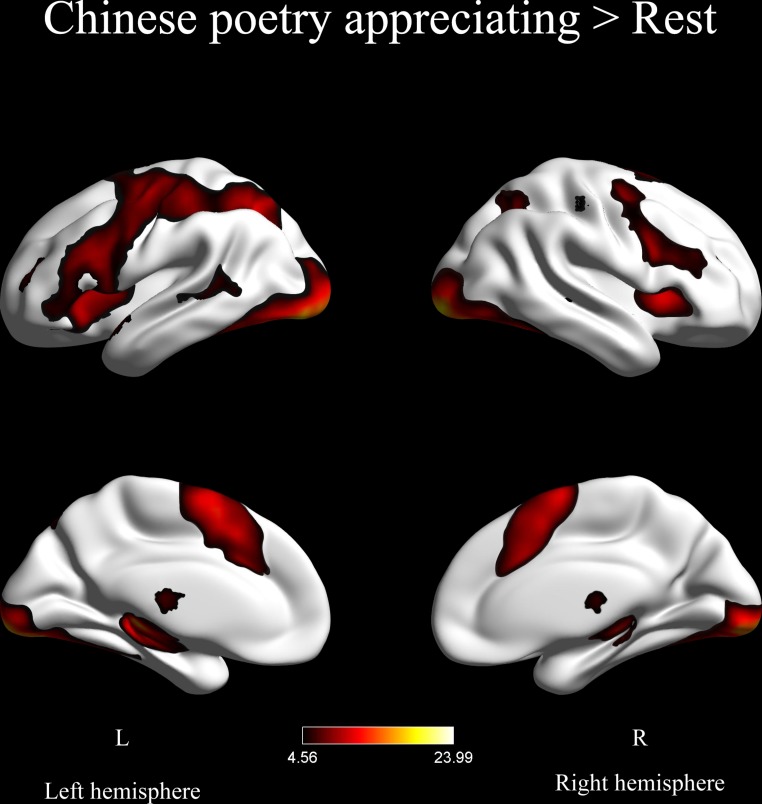
Areas of brain activation during the appreciation of Chinese poetry (*p* < 0.001, FDR corrected).

The areas of brain activation during the appreciation of Chinese poetry are depicted in **Figure 4**. Significant activation was found in the bilateral inferior frontal gyri, bilateral inferior temporal gyri, left inferior parietal gyrus, left parahippocampal gyrus, left lingual gyrus, bilateral occipital lobes, left fusiform, bilateral insula, left supplementary motor area (SMA), and left precentral gyrus. Appreciating Chinese poetry induced massive activation of bilateral parts of the cerebral hemispheres.

#### Differences Between the Appreciation of Chinese Poetry and That of Prose

**FIGURE 5:**
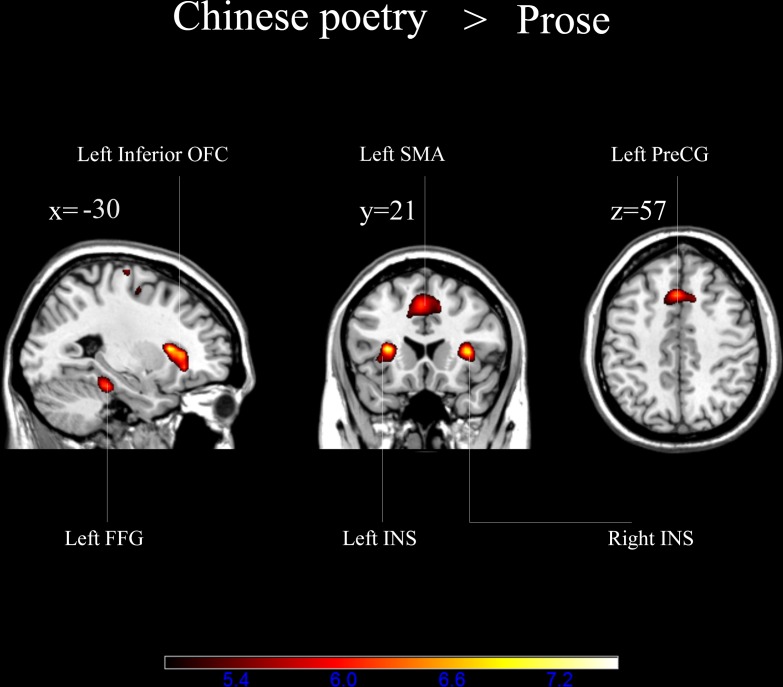
Brain activation during the appreciation of Chinese poetry vs. that of prose (*p* < 0.001, FDR corrected). OFC, orbitofrontal cortex; INS, insula; FFG, fusiform gyrus; SMA, supplementary motor area; PreCG, precentral gyrus.

To investigate the neural substrates of the feeling of beauty induced by appreciating Chinese poetry, a subtraction analysis comparing the appreciation of Chinese poetry to that of prose was conducted to determine potential differences in blood oxygenation level dependent signal levels during the reading. There were significantly higher activation levels in the left inferior OFC, left fusiform, bilateral insula, left SMA, and left precentral gyrus (**Figure 5** and **Table 1**).

**Table 1:** Areas of brain activation during the appreciation of Chinese poetry vs. that of prose (*p* < 0.001, FDR corrected).

Brain regions	Hemisphere	MNI coordinates			*t*-score	Cluster size
				
		*x*	*y*	*Z*		
Inferior OFC	L	-30	29	-6	5.82	35
Insula	L	-30	21	9	7.38	100
	R	33	24	6	7.15	66
Fusiform	L	-30	-36	-18	6.70	87
SMA	L	-3	21	45	6.42	125
Precentral gyrus	L	-33	-6	57	6.24	212


*FDR, false discovery rate; MNI, Montreal Neurological Institute; OFC, orbitofrontal cortex; SMA, supplementary motor area.*

## Discussion

Chinese poetry is a special form of poetry and is deeply loved by readers worldwide. Individuals appreciate Chinese poetry in a subjective and engaged manner while experiencing the mood of the work, the feelings it evokes, and the poetic imagery it implies. Poetry, as the interlude of language and music, has phonological features such as those found in music. These phonological features can influence the appreciation of poetry (Lerdahl, 2001; Li and Yang, 2010). The meter and rhyme of poetry also have an impact on esthetic appreciation (Obermeier et al., 2013). Individuals are able to feel beauty during the appreciation of poetry. This has been demonstrated by our behavioral results. Participants were requested to rate the beauty induced by appreciating Chinese poetry and that induced by prose on a scale ranging from 1 (no sense of beauty) to 4 (great sense of beauty). When reading Chinese poetry, the participants felt high levels of beauty (*M* = 3.58, standard deviation = 0.64) and assigned a rating 4 to 65.43% of the stimuli and a rating of 1 to only 0.43% of the stimuli. In contrast, the participants reading prose felt low levels of beauty (*M* = 1.33, standard deviation = 0.70) and assigned a rating of 1 to 78.43% of the stimuli, and a rating of 4 to only 2% of stimuli (**Figure 3**). The behavioral results thus indicate that the participants felt beauty when reading Chinese poetry and that the beauty felt was significantly higher than that felt during the reading of prose. The feeling of beauty is one of the reasons that we enjoy reading Chinese poetry, although the processes that occur in our brains during the appreciation of Chinese poetry and why we feel beauty while appreciating Chinese poetry are still unknown.

Previous researchers have explored the secrets of poetry at a neural level, e.g., Al’tman la (1995). However, earlier studies were devoted to analysis of the mental state under poetic inspiration and produced no consistent conclusions regarding the neural mechanisms underlying the appreciation of poetry. Here we were interested in brain activation related to the appreciation of Chinese poetry. Our analysis of the blood oxygenation level-dependent signals during the appreciation of Chinese poetry revealed significant activation in large parts of the brain (see **Figure 4**). Scattered regions in both cerebral hemispheres were activated. Some of the activated regions were related to visual stimulus perception, sentence reading, and semantic processing. This finding is partly in accordance with previous research on the esthetic appreciation of other forms of literature. Bohrn et al. (2013) have reported that large parts of the left frontal lobe, bilateral middle temporal gyri, bilateral superior temporal gyri, bilateral occipital lobes, and bilateral precentral gyri were activated during the reading of proverbs. The left inferior frontal gyrus, the bilateral occipital lobes, and the left precentral gyrus are areas that were activated in both studies. Zeman et al. (2013) showed that the reading of both poetry and prose can lead to activation of the left inferior frontal gyrus, left precentral gyrus, left middle temporal gyrus, bilateral middle occipital gyri, and left mid-fusiform cortex. The same regions were also activated in our study. Specifically, we observed activation of the left inferior frontal gyrus, left precentral gyrus, bilateral occipital gyri, and left fusiform. These findings suggest that reading literature or poetry of any kind leads to the activation of similar neural substrates. This supports our hypothesis H1. Among these related regions, the bilateral occipital lobes were strongly activated when reading Chinese poetry. This indicates that the brain can receive visual stimulation from the poetry and induce intense visual attention to beautiful visual objects (Cela-Conde et al., 2004). The activations of the bilateral inferior frontal gyri, bilateral inferior temporal gyri, left inferior parietal gyrus, left parahippocampal gyrus, and left lingual gyrus have been reported during sentence reading and semantic processing (Mechelli et al., 2000; Keller et al., 2001; Binder et al., 2009). Activation of the bilateral middle frontal gyri is related to rhymes and tones in the reading of Chinese text (Gandour et al., 2003). These findings indicate that the process of appreciating Chinese poetry involves at a minimum the perception of the visual words, sentence reading, and the semantic interpretation of the poetry.

Appreciating Chinese poetry led to the activation of other parts of the brain in addition to those described above. These regions were differentially activated during the appreciation of Chinese poetry vs. the prose selections. The contrast between the appreciation of poetry and that of prose was reflected in the activation of a distributed network including the left inferior OFC, bilateral insula, left fusiform, left SMA, and left precentral gyrus (**Figure 5** and **Table 1**). Among these regions, the OFC and the insula are key substrates of the feeling of beauty. These two regions have been identified as important “esthetic centers” in the human brain (Brown et al., 2011). Brown et al. (2011) carried out the most comprehensive analysis of neuroesthetic processing by carrying out voxel-based meta-analyses of 93 neuroimaging studies of positive-valence esthetic appraisal across four sensory modalities. The results of the above investigation indicate that the most concordant area of activation across all four modalities is the right anterior insula. The authors of the above study the co-activation of the sensory-specific regions of the OFC and a supermodel area located in the anterior insula are involved in the overall process of esthetic appreciation. Furthermore, Blood and Zatorre (2001) found that the OFC and the insula took part in the appreciation of music and were related to musical “chills,” which is a phenomenon that occurs during esthetic appreciation. In addition, Zeman et al. (2013) reported similar results using the experimental poetic material.

We observed differences in the activation of the left inferior OFC while the participants appreciate Chinese poetry vs. prose. The OFC, and especially the medial OFC, has been shown to be important for the feeling of beauty by previous studies on different art forms (Blood and Zatorre, 2001; Kawabata and Zeki, 2004; Jacobsen et al., 2006; Di Dio et al., 2007; Kirk et al., 2009b; Zhang et al., 2016). Kawabata and Zeki (2004) suggested that activation of the OFC correlates with judgments of beauty and ugliness. Kirk et al. (2009a) compared experts and non-experts in their appreciation of architectural stimuli and concluded that the response profile of the medial OFC in both groups had a positive linear correlation with esthetic ratings. The OFC is sensitive to the magnitude of the esthetic value. This is supported by studies of reward processing, which show that the relative reward values of the stimuli are reflected by the amplitudes of neural activity in the OFC (Kringelbach, 2005). The hypothesis of valence processing, which was introduced by Kringelbach (2005), states that the medial OFC is associated with positive valence, while the lateral OFC is involved in negative valence. As mentioned above, the OFC has an important role in the feeling of beauty. We thus assume that it also plays a key role in the feeling of beauty induced by Chinese poetry.

Activation of the bilateral insula in our study indicates that it may be involved in the generation of esthetic emotions when the participant is appreciating the beauty of Chinese poetry. This result agrees very well with previous studies of esthetics in other domains. Di Dio et al. (2007) have shown that the right anterior insula is activated when participants view objectively beautiful sculptures when compared to sculptures modified to be less proportional. The authors propose that the positive feeling elicited by viewing the canonical sculptures is mediated by a cortical network involving the anterior insula. Cupchik et al. (2009) have reported that esthetic perception activates the bilateral insula. They believe that the bilateral insula play important roles in esthetic emotions. Two other large-scale meta-analyses of neuroimaging studies on esthetic appreciation also concluded that the anterior insula is a key structure in experiencing esthetic emotions. The insula, and especially the anterior insula, is known to play a critical role in emotional processing (Craig, 2010). The left side of the insula is associated with the evaluation of positive emotions, while the right side is associated with the evaluation of negative emotions (Craig, 2005, 2010; Tsukiura and Cabeza, 2011). Leder et al. (2004) have suggested that the insula is involved in “continuous affective evaluation”. Compared to prose, Chinese poetry contain more complex and abundant emotions. Therefore, appreciating the beauty of Chinese poetry produces many explicit esthetic emotions, which probably involve the function of the insula. We found significantly higher activation of the bilateral insula in participants appreciating Chinese poetry vs. those reading prose.

In addition to the left inferior OFC and the bilateral insula, the left fusiform, the left precentral gyrus, and the left SMA were significantly more highly activated during the reading of poetry vs. that of prose. Similar results have been reported in previous studies of esthetics using words as stimuli (Bohrn et al., 2013; Zeman et al., 2013; Hsu et al., 2014; Zhang et al., 2016). These areas are thus probably related to the process of reading and understanding of words and literature. The left fusiform is associated with reading single words, and is often described as the visual word form area (Jobard et al., 2003). Some researchers also hypothesize that this area is dedicated to determining and understanding word meanings (Devlin et al., 2006). The left precentral gyrus had heightened activation in response to Chinese poetry. This activation may be linked to motor activation via the processes of understanding (Pulvermuller et al., 2005). Activation of these regions is mainly involved in the perception of esthetics.

Based on the discussion above, the fMRI results confirm our hypothesis H2. We compared brain activity patterns during the appreciation of Chinese poetry to those during the reading of prose. We found significant activation in specific brain regions (e.g., OFC and insula) related to the appreciation of beauty. This result is in accordance with those of studies of brain regions related to the feeling of beauty induced by other art forms.

Here, we investigated the neural correlates of feeing beauty induced by the appreciation of Chinese poetry. Appreciation of Chinese poetry is a very complicated and complex process, and involves the perception of words, sentence reading, comprehension of poetry, and the processing of esthetic emotion. Appreciating Chinese poetry thus requires different brain regions to work together. The bilateral inferior frontal gyri, bilateral inferior temporal gyri, left inferior parietal gyrus, left parahippocampal gyrus, left lingual gyrus, bilateral occipital lobes, left fusiform, bilateral insula, left SMA, and left precentral gyrus were more strongly activated during the appreciation of Chinese poetry. Our behavioral results indicate that reading Chinese poetry leads to much higher feelings of beauty than reading prose. Investigating the differences between the appreciation of Chinese poetry and that of prose can thus help us to better understand the neural substrates of beauty when appreciating Chinese poetry. We found increased activation in the left inferior OFC, left fusiform, bilateral insula, left SMA, and left precentral gyrus during the appreciation of Chinese poetry when compared to the reading of prose. These findings suggest that some of the neural correlates associated with feelings of beauty induced by Chinese poetry are the same as those activated by stimuli from other esthetic domains. While the present research is a tentative exploration, our findings suggest the possibility that the above regions are closely associated with feelings of beauty induced by Chinese poetry. The concrete mechanisms underlying the activation of these regions and their association with feelings of beauty induced by Chinese poetry merits further exploration in future studies.

## Ethics Statement

This study was carried out in accordance with the recommendations of the Human Ethics Committee of the Southwest University for the Brain Mapping Research with written informed consent from all subjects. All subjects gave written informed consent in accordance with the Declaration of Helsinki. The protocol was approved by the Human Ethics Committee of the Southwest University for the Brain Mapping Research.

## Author Contributions

All the authors worked together to design the experiment, prepared materials, conducted the experiment, and finally they analyzed the data and finished this paper. All the authors agreed that the final version of the manuscript is suitable for submission and approved to take responsibilities for every aspect of the whole work.

## Conflict of Interest Statement

The authors declare that the research was conducted in the absence of any commercial or financial relationships that could be construed as a potential conflict of interest.

## Footnotes

1 Our research focuses on the appreciation of Chinese poetry while reading silently by oneself. Reading aloud or being read to by others is not considered here.

2 www.fil.ion.ucl.ac.uk/spm
